# When crystals flow

**DOI:** 10.1126/sciadv.adg8865

**Published:** 2023-05-10

**Authors:** Chien-Hua Tu, Martin Steinhart, Rüdiger Berger, Michael Kappl, Hans-Jürgen Butt, George Floudas

**Affiliations:** ^1^ Max Planck Institute for Polymer Research, 55128 Mainz, Germany.; ^2^ Institut für Chemie neuer Materialien, Universität Osnabrück, D-49069 Osnabrück, Germany.; ^3^ Department of Physics, University of Ioannina, 45110 Ioannina, Greece.; ^4^ University Research Center of Ioannina (URCI) - Institute of Materials Science and Computing, 45110 Ioannina, Greece.

## Abstract

Semicrystalline polymers are solids that are supposed to flow only above their melting temperature. By using confinement within nanoscopic cylindrical pores, we show that a semicrystalline polymer can flow at temperatures below the melting point with a viscosity intermediate to the melt and crystal states. During this process, the capillary force is strong and drags the polymer chains in the pores without melting the crystal. The unexpected enhancement in flow, while preserving the polymer crystallites, is of importance in the design of polymer processing conditions applicable at low temperatures, e.g., cold drawn polymers such as polytetrafluoroethylene, self-healing, and in nanoconfined donor/acceptor polymers used in organic electronics.

## Teaser

Semicrystalline polymers can be made to flow at temperatures below their melting point in pores by capillary action.

## INTRODUCTION

Perfect crystals at zero temperature do not flow. There is a scientific prejudice that crystals do flow under any conditions. However, Heraclitus, some 2500 years ago, proposed that “everything flows.” Since then, there are several examples showing the flow of crystalline materials. As an example, Rosenhain and Ewen (*1*), more than 100 years ago, suggested the flow of cast iron in terms of the flow of metal grains surrounded by a thin amorphous layer, being analogous to a undercooled liquid (*2*). Recent molecular dynamics simulations (*3*) have confirmed these ideas and further suggested the importance of the complex grain boundary “fluid” on the mechanism of plastic deformation. Along the same lines, Earth’s inner core has been proposed to be composed of iron in a bcc crystalline state that exhibits large-scale anisotropic structures (*4*). Similarly, the core of some other planets (Neptune and Uranus) has been suggested to be composed of superionic crystalline water (*5*). To generate their magnetic field, the cores of these planets must flow. Our very existence might well be associated with the flow of these crystalline materials. Faraday (*6*), some 170 years ago, suggested that the “slipperiness” of ice below 273 K was due to the formation of a fluid-like interfacial crystal ice. Much later, Tammann suggested that crystalline materials become “mobile” or “active” at temperatures well below their melting temperature, *T*_m_, e.g., at temperatures as low as ~2/3*T*_m_ (*7*, *8*). Another class of crystalline materials that exhibit fluid-like mobilities is the “superionics” of importance for energy applications (*9*). The latter have a structure that resemble to crystals, yet they show fast-ion diffusion as in liquids. Examples include the component of reactor fuel, crystalline UO_2_, where the ionic O^2−^ mobility can be extraordinary high that gives rise to some safety issues (*10*). Other examples are the fast ion diffusion in inorganic (*11*) and, more recently, in organic (*12*) solid-state electrolytes.

Semicrystalline polymers are also solids that do not flow under normal conditions. They are composed of polymer chains embedded in a unit cell that are further folded in a lamellar morphology comprising chains participating in crystalline and amorphous domains (*13*, *14*). The characteristic domain spacing—depending on the chain length and annealing conditions—is in the range from 10 to 50 nm. However, recent reports argued that polymer glasses can gain mobility when deformed (*15*). Likewise, polymer melts of high viscosity are capable to penetrate narrow pores with a lower viscosity than in bulk (*16*, *17*). In all cases, polymer adsorption plays a dominant role (*18*–*20*). Here, we show that even semicrystalline polymers flow. When placed on top of nanoporous templates, they imbibe the pores by capillary action.

Two semicrystalline polymers were used; poly(ethylene oxide) (PEO1k, PEO8k, and PEO500k with respective molar masses and dispersities: *M*_n_ = 825, 8090, and 398,000 g/mol, *Ð* = 1.23, 1.13, and 1.21) and poly(ε-caprolactone) (PCL5k; *M*_n_ = 5370 g/mol, *Ð* = 1.81) with molecular characteristics shown in table S1. Main results shown here relate to PEO. Results for PCL5k can be found in the Supplementary Materials. Self-ordered nanoporous alumina templates [anodic aluminum oxide (AAO)] were prepared following the literature procedures (*21*, *22*). AAO contains arrays of parallel cylindrical nanopores uniform in length (100 μm) and diameter (pore diameters ranging from 25 to 400 nm).

## RESULTS

**Fig. 1.**
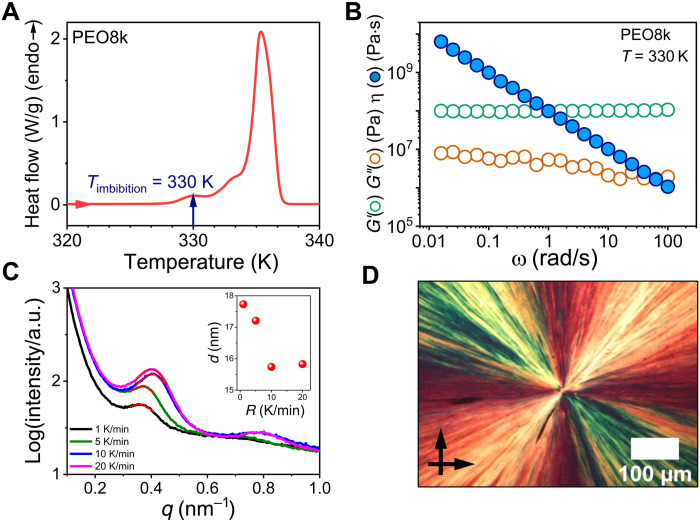
Bulk PEO8k thermodynamics, rheology, and morphology. (**A**) Differential scanning calorimetry (DSC) heating curve (rate = 10 K/min) showing (apparent) melting at 335 K. The imbibition temperature (*T* = 330 K) is indicated with an arrow. (**B**) Storage modulus (green circles), loss modulus (red circles), and viscosity (blue spheres) measured at the imbibition temperature (*T* = 330 K) measured in rheology. (**C**) Small-angle x-ray scattering (SAXS) curves of PEO8k obtained at ambient temperature following slow cooling from the melt. The lamellar domain spacing is shown in the inset for different cooling rates, *R*. a.u., arbitrary units. (**D**) Spherulitic morphology obtained by polarizing optical microscopy (POM) with crossed polars at ambient temperature following slow cooling from the melt.

The thermodynamic, structural, and rheological properties of bulk PEO8k are compiled in Fig. 1. The differential scanning calorimetry (DSC) heating curve (Fig. 1A) shows a major melting peak at a temperature of around 335 K. Some minor melting peak at 330 K corresponds to the melting of a minor lamellae (the heat of fusion of the minor peak at 330 K is 5 J/g as compared to the 104 J/g for the main melting peak at 335 K). The degree of crystallinity, *X*_c_, of the PEO8k was high (*X*_c_ = 70 ± 2%) [*X*_c_ = Δ*H*/Δ*H*_∞_, where Δ*H* is the measured heat of fusion and Δ*H*_∞_ is the estimated enthalpy of fusion for 100% crystalline PEO (197.8 J/g)] (*13*). The imbibition temperature was chosen at 330 K, i.e., well below the main melting peak at 335 K. Figure 1B shows the viscoelastic behavior of PEO8k measured at the imbibition temperature (*T* = 330 K). As expected, the storage (*G*′) and loss (*G*″) moduli are independent of frequency exhibiting elastic response (with *G*′ > *G*″). A zero shear viscosity was never reached. These data confirm that the PEO8k film on top of AAO template was in the semicrystalline state. The domain spacing, *d*, of PEO8k crystals obtained from small-angle x-ray scattering (SAXS) (Fig. 1C) was in the range from 15 to 18 nm depending on the cooling rate (*R*). Crystalline lamellae were far smaller than the pore size (400 nm in this case). The superstructure formation in bulk PEO8k was studied by polarizing optical microscopy (POM) in a film that was slowly cooled from the melt to ambient temperature. Figure 1D depicts a single spherulitic superstructure, expected from the low nucleation density of PEO. The single spherulite in POM is distinctly different from the high nucleation density in PCL (fig. S2D). The vastly different nucleation densities of PEO and PCL were discussed (*23*, *24*) in terms of the synthesis method: The use of a catalyst in PCL promotes the heterogeneous nucleation. In contrast, PEO was the synthesized from the gas phase in the absence of catalyst.

**Fig. 2.**
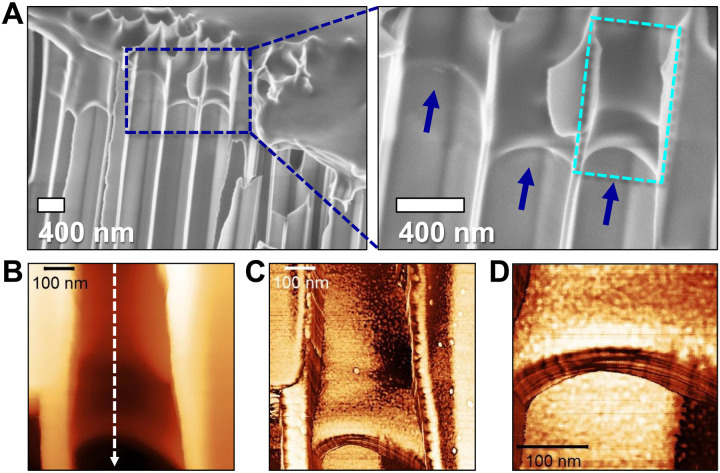
Imbibition of PEO8k in nanopores revealed by scanning electron microscopy (SEM) and atomic force microscopy (AFM). (**A**) Left: SEM image from a fractured surface of anodic aluminum oxide (AAO) having nanopores with a diameter of 400 nm infiltrated with PEO8k at 330 K for 28 days. Right: A zoom-in to the blue rectangular dashed area in left. Blue arrows indicate the meniscus. (**B**) AFM two-dimensional height image corresponding to the cyan rectangular dashed area of (A, right) (**C**) AFM two-dimensional image to the same area as (B). (**D**) A zoom-in AFM two-dimensional image to the meniscus region.

Following a 28-day imbibition of PEO8k in AAO templates with a pore diameter of 400 nm, the sample was cooled to ambient temperature, and scanning electron microscopy (SEM) and atomic force microscopy (AFM) were used to characterize AAO cross sections (Fig. 2A). There were two important findings from SEM: (i) PEO8k clearly enters into the nanopores at a temperature of 5 ± 0.2 K below the melting temperature with imbibition lengths in the range from 400 to 800 nm. (ii) There exists a meniscus at the growth front. The meniscus provides a direct proof of capillary force. Besides, the concave meniscus implies that the adhesion force between polymer and AAO wall is stronger than the cohesive force between the polymer chains. Figure 2 (B to D) depicts the AFM image taken from the same region as in Fig. 2A. The image confirms the presence of PEO within the nanopores and further shows the formation of the meniscus. It has been argued that for amorphous polymers above the glass temperature or molten polymers, a precursor film of a polymer with thickness of few nanometers moves ahead of the meniscus (*25*). The AFM results demonstrate the absence of this layer. With respect to the surface morphology, a strong contrast exists for PEO8k chains located at the top of the film and the ones located inside the nanopores (the former exhibit long fibrillar structures; fig. S3).

The successful imbibition was demonstrated for PCL5k as well (fig. S4). Both cases prove that capillary force is strong enough to drag the polymer crystals in the nanopores at a temperature below the melting point, *T*_m_. AFM characterization also applied to the confined PCL5k (figs. S4 and S5). In contrast to the relatively smooth profile in PEO8k, PCL5k shows abundant grain structures densely distributed along the pores. The multigrain structure inherits from the high nucleation density in the bulk sample (fig. S2). It was suggested (*26*) that the origin of the different morphologies is the difference in intracrystalline chain diffusion between PEO (known as crystal mobile) and PCL (crystal fixed).

### Polymer morphology inside nanopores

**Fig. 3.**
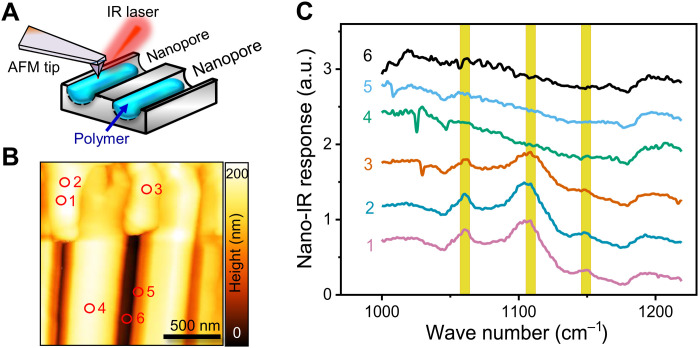
Nano–infrared (IR) reveals the semicrystalline nature of the polymer in the nanopores. (**A**) Schematic arrangement of the sample for the nano-IR measurements. After scanning the edge of the anodic aluminum oxide (AAO) surface, the atomic force microscopy (AFM) tip is positioned on the polymer or on the AAO surface. Then, the wavelength of the IR laser is tuned. The vibrational amplitude of the AFM tip corresponds to the nano-IR response of the selected position, respectively. (**B**) Topographic image of the AAO nanoporous sample filled by PEO8k. We selected three positions on the PEO (#1, #2, and #3) and three positions on the AAO (#4, #5, and #6), respectively, and recorded IR spectra. (**C**) Corresponding IR spectra taken at the marked positions in (B). For positions #1 to #3, absorption peaks at 1061, 1108, and 1149 cm^−1^ are present (in yellow) that are absent for positions #4 to #6. Peaks at 1061, 1108, and 1149 cm^−1^ are typical for semicrystalline PEO (*27*). The latter three peaks are also present on PEO films (fig. S1).

In addition to topography characterization (AFM), we performed nano–infrared (IR) microscopy in the photo-induced force microscopy (PiFM) mode. To obtain images of the surface topography, we excited the second eigenmode of the cantilever resonance frequency and kept a constant amplitude of vibration with an electronic feedback circuit. The first eigenmode of the cantilever resonance frequency was used to record the IR light–induced forces. The incoming IR light was modulated at the difference frequency between the first and second eigenmodes. This difference frequency was fine-tuned to a maximum response amplitude at the first eigenmode, while the tip was engaged on the surface. Spectra in PiFM mode were recorded by ramping the wave number of the IR light from 850 to 1850 cm^−1^ (Fig. 3) Control experiments of semicrystalline PEO8k were made on a film deposited on a glass slide (fig. S1). The nano-IR measurements showed three noticeable absorption peaks at 1149, 1100, and 1061 cm^−1^ characteristic of the semicrystalline PEO. The peak at 1061 cm^−1^ reflects CH_2_ rocking with CO stretching, whereas the remaining peaks in the region at 990 to 1200 cm^−1^ associate with CH_2_ and CO rocking/stretching (*15*). In the melt, the fine vibrational structure is replaced by a large band. The CO band is wider with a broad maximum at ~1109 cm^−1^. Subsequently, IR-AFM was used to characterize the state of the polymer inside the pores (*27*). Different imaging positions were selected corresponding to the polymer and the AAO channels. Intense adsorption peaks at 1061, 1108, and 1149 cm^−1^ clearly demonstrate the state of semicrystalline PEO within the nanopores.

What is the effective viscosity of the polymer during imbibition? We can estimate the effective viscosity, η_eff_, using the Lucas-Washburn equation applicable to Newtonian liquids (*28*–*30*)

$$h(t)={\left(\frac{\gamma R\cos\theta }{2\eta }\right)}^{1/2}\sqrt{t}$$

Here, *h*(*t*) is the imbibition length (*h* = 800 nm), γ is the surface tension (~0.029 N/m), θ is the advancing contact angle (θ ~ 44°) (*18*), *R* is the pore radius (200 nm), and *t* is the imbibition time (*t* ~ 2 × 10^6^ s). The equation reflects the balance between two opposite forces: the capillary force that drives the imbibition and the viscous force that provides the friction against imbibition (*31*). From the imbibition length, we obtain an effective viscosity of 8 × 10^9^ Pa·s. This value should be contrasted with the PEO8k bulk viscosity in the crystalline and melt states, respectively. At a shear rate of $\dot{\gamma }=\nu /R≅\;$10^−6^ s^−1^ (*ν* is the imbibition speed obtained as ν = *dh*/*dt*~ 3 × 10^−13^ m/s) applicable during imbibition, the bulk viscosity at 330 K (crystalline state) is much higher than 10^10^ Pa·s (Fig. 1B). On the other hand, the zero-shear viscosity of the melt state, η_0_, is only 2 × 10^3^ Pa·s (fig. S6). Thus, the semicrystalline polymer flows within the nanopores with a viscosity between the crystalline and melt states, i.e., η_melt_(2 × 10^3^ Pa·s) < η_eff_ (8 × 10^9^ Pa·s) < η_crystal_ (higher than 10^10^ Pa·s). It suggests that a fraction of crystals are molten during the imbibition process. This is likely, given that the force to pull a single PEO chain out of its single crystal is *F* ~ 40 pN (*32*), producing an energy (*W* = *F* × *L*, where *L* = *n* × 0.358 nm and *n* is the degree of polymerization) of 3 × 10^−18^ J, i.e., a small fraction of the total energy needed to melt the entire crystal (1 × 10^−11^ J).

Furthermore, we address the possibility that the magnitude of the capillary force is high enough to actually melt the crystals during flow. In this scenario the chains are molten by the capillary action on entering the pores and the polymer re-crystallize within the pores. To this end we employ *F*_viscous_ = 8πη_eff_*hv*, for the drag force that results to 5 × 10^−8^ N with a corresponding energy of $W_{\mathrm{drag}}=\int_{0}^{h}F_{\mathrm{drag}}dx$ ~ 4 × 10^−14^ J. This energy is a minor fraction of the energy needed to melt the crystals (1 × 10^−11^ J for the main melting peak at 335 K). Hence, the polymer chains enter the pores by capillary action in their folded semicrystalline state.

One could argue that the reason for the reduced viscosity of the semicrystalline polymers is the enhanced segmental relaxation under confinement. A faster segmental dynamics has been observed for a number of confined polymers (*30*, *33*). To explore this possibility, we have used dielectric spectroscopy (DS) to investigate the segmental dynamics of the confined semicrystalline polymers. The method follows the segmental dynamics of the amorphous domains that are confined by the crystalline domains. As shown in fig. S7, a single dielectric relaxation is observed in the vicinity of the bulk glass temperature . The temperature dependence of the characteristic frequency at maximum loss follows an Arrhenius dependence as $f=f_{0}\exp\left(-\frac{E}{RT}\right)$ for confined PEO. Here, *f*_0_ is a characteristic frequency in the limit of very high temperatures, and *E* is the activation energy. Alternatively, for confined PCL, it follows a Vogel-Fulcher-Tammann behavior according to $f=f_{0}\exp\left(-\frac{B}{T-T_{0}}\right)$. Here, *B* is the activation parameter, and *T*_0_ is the “ideal” glass temperature located below the DSC *T*_g_. For the glass temperature, we use the usual definition as the temperature where the segmental relaxation time equals 100 s. We should mention that the uncertainty in the determination of *T*_g_ is high under conditions of confinement for two reasons: First, both polymers are semicrystalline, and the glass temperature refers only to the amorphous segments (i.e., the dielectric signal is weak). Second, under confinement, all dynamic processes become broad increasing the uncertainty. Nevertheless, in both cases, the glass temperature is reduced form the bulk by about 9 ± 4 K and 5 ± 3 K, respectively, for PEO (*T*_g_^bulk^ = 206 K) and PCL (*T*_g_^bulk^ = 201 K). These small reductions in the glass temperature cannot account for the reduced effective viscosity of the semicrystalline polymer since the imbibition temperature is ~110 K above *T*_g_.

### Mechanism of imbibition and relevant time scales

**Fig. 4.**
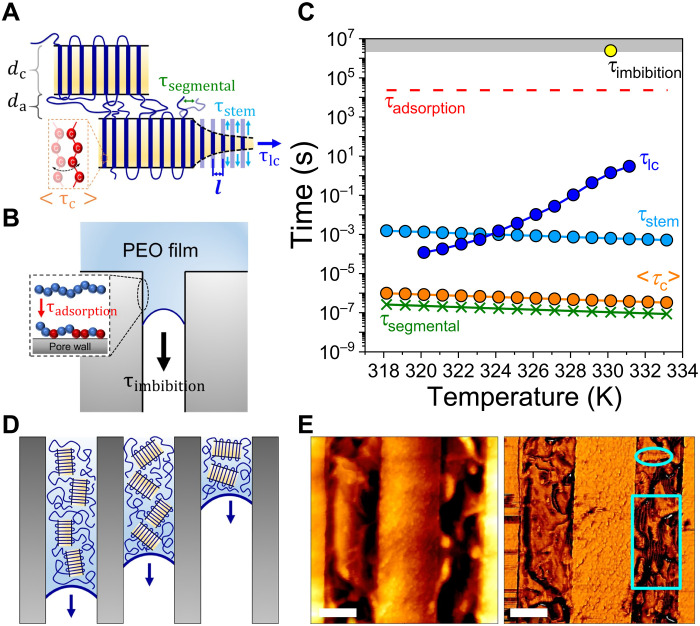
Molecular mechanism and associated time scales during imbibition of semicrystalline polymers in nanopores. (**A** and **B**) Hierarchical structures and associated kinetics pertinent to semicrystalline polymers: (A) Organization of polymer chains into ordered lamellae involves the movement of segments within the crystalline (*d*_c_) and amorphous (*d*_α_) regions. Four processes are defined as follows: τ_stem_ represents the diffusion time of PEO chains in the length scale of the crystalline domain (*d*_c_); τ_lc_ illustrates the growth time of a unit crystal lamellae; <τ_c_> depicts the switching time of two helical defect jumps within the crystal; τ_segmental_ represents the segmental dynamics within the amorphous regions. *l* represents the intermolecular distance (*l* = <*a* + *b*>/2; *a* and *b* are the crystal unit cell parameters along *x* and *y* axes, respectively). (B) Imbibition of polymer chains (τ_imbibition_) proceeds via adsorption with a characteristic time τ_adsorption_. The anchored units on the pore walls are indicated with the red color. (**C**) Characteristic time scales and their temperature dependence: τ_imbibition_ (yellow sphere), τ_adsorption_ (red dashed line), τ_lc_ (blue spheres), τ_stem_ (cyan spheres), <τ_c_ > (orange spheres), and τ_segmental_ (green crosses). The original data are provided in table S1. (**D**) The effect of lamellar orientation with respect to the pore axes on the variable penetration lengths. (**E**) AFM height (left) and phase (right) images reveal well-oriented crystalline (PCL) lamellae inside nanopores (cyan framed regions). Scale bars, 200 nm.

Understanding the mechanism of imbibition from the semicrystalline state requires addressing the pertinent dynamics in the crystalline and amorphous domains. Figure 4 compiles six dynamic processes pertinent to semicrystalline PEO8k. Four processes are acting within the amorphous and crystalline regions (Fig. 4A). In the amorphous domain, the dynamics is governed by the segmental relaxation. In the crystalline domain, three processes are identified by nuclear magnetic resonance and POM (*26*, *34*): (i) local helical defect jumps within the crystal (time <τ_c_>), (ii) the diffusion of PEO chains over a length scale of lamellar thickness (*d*_c_) (time τ_stem_), and (iii) the growth of a unit crystal lamellae (time τ_lc_) (obtained from POM). Processes (i) and (ii) reflect the so-called intracrystalline chain diffusion, which is particularly important for crystal-mobile polymers such as PEO. In the largest field of view (Fig. 4D), the main focus of this study—imbibition of crystals—also involves the diffusion of entire crystallites, depicted as τ_imbibition_. Moreover, the adsorption of polymer chains during imbibition is described by τ_adsorption_. Details on these characteristic times are provided in figs. S8 and S9. Figure 4C shows the temperature dependence of the characteristic times. The fastest dynamics here belongs to the segmental relaxation of polymer segments within the amorphous region, i.e., τ_segmental_ (green crosses in Fig. 4C). The corresponding dynamics inside the crystal, <τ_c_>, during which two helical defect jumps switch back and forth, is about one decade slower. The other two crystal time scales, τ_stem_ and crystal growth (τ_lc_) processes, are slower, within the millisecond to 10-s time scales and even cross at 326 K. The imbibition of PEO crystals and the adsorption of polymer chains are, expectedly, the two longest processes (yellow sphere and red dashed line). The latter (adsorption) reflects the continuous exchange between polymer segments/chains at the vicinity of pore walls in which unfavorable conformations (loops, trains, and tails) and severe topological constraints (i.e., entanglements) are formed (*35*, *36*). The imbibition time scale is 6 to 12 orders of magnitude slower than the crystal time scales (<τ_c_>, τ_stem_, and τ_lc_), suggesting slow cooperative diffusion of crystalline lamellae as the main mechanism.

How does the molar mass of the polymer affect the imbibition process? The results reveal that the imbibition speed is controlled by the molar mass and, in particular, by the chain folding process. PEO1k (*M*_n_ = 825 g/mol, *Ð* = 1.23) enters the pores with a higher speed as compared to PEO8k (fig. S10). The lamellar structure of PEO8k (chain dimensions of 2*R*_g_ ~ 7 nm; *R*_g_ is the radius of gyration and the domain spacing is 16 nm) is composed of folded PEO chains (with integral folding of 2) within the crystal domain of 12.6 nm. Hence, a single chain binds two crystalline domains. On the other hand, PEO1k crystallizes in a fully extended configuration within a single-crystalline domain. The latter effectively comprise brushes that are lubricated and can easily slide one another during the flow. Contrast this with the situation in higher molar mass polymers. There, a single chain “binds” several crystalline domains, making it more difficult for a chain to exit the crystal. PEO with a higher molar mass, PEO500k (*M*_n_ = 398000 g/mol, *Ð* = 1.21), could not enter the same nanopores (fig. S10).

Last, we address the distribution of penetration lengths for various nanochannels (Fig. 2A and fig. S4) despite the identical conditions. This effect reflects the different lamellar orientations with respect to the nanotube axis. When lamellae find the pore opening with the “correct” orientation (i.e., with their long “growth” axis parallel to the pore axis), they can easily penetrate as opposed to lamellae with the “wrong” orientation (Fig. 4D). The longest penetration lengths are composed of lamellae with the correct, i.e., the parallel orientation (Fig. 4E).

## DISCUSSION

The SEM, AFM, and nano-IR results demonstrated that semicrystalline polymers can flow within nanopores via capillary action. Evidently, capillary action drives the polymer adsorption process (fig. S11). Although extremely slow, the successful imbibition shows that the capillary force is strong enough to drag polymer crystallites into pores without melting the crystals. The small reduction in the liquid-to-glass temperature by confinement cannot account for the large decrease in viscosity. Shorter polymer chains in the absence of chain folding accelerate this process because crystalline domains effectively comprise lubricated brushes. The unexpected enhancement in flow while preserving the polymer crystallites is of importance in the design of polymer processing conditions applicable at low temperatures. Applications include cold flow and subsequent bonding of polymers to ceramics or metals via porous adhesion-mediated layers under conditions where polymer degradation is prevented. Another example is cold drawn polytetrafluoroethylene yarns. The latter are used in implantable vascular prostheses and show excellent abrasion resistance strength and lubrication characteristics that closely resemble to a natural body lumen (*37*). Moreover, slow capillary flow of confined semicrystalline donor/acceptor polymers and ferroelectric materials (*38*–*40*) used in organic electronics will result to time-dependent properties affecting their electronic/physical properties.

## MATERIALS AND METHODS

### Materials

Two semicrystalline polymers with variable molar masses were used in this study: PEO and PCL. Their molecular characteristics are given in table S1.

### AAO templates and methods of infiltration

Self-ordered nanoporous AAO templates were fabricated via an electrochemical method, as reported earlier (*41*–*43*). The pore diameter and pore length were 400 nm and 100 μm, respectively. Before infiltration, all AAO templates were annealed in a vacuum oven at 423 K for 8 to 10 hours. Two thin films of PEO8k and PCL9k were prepared by hot pressing above their melting temperature. The films were subsequently cooled slowly to ambient temperature. Subsequently, they were deposited on top of AAO template inside a vacuum oven at preset temperatures of 330 and 323 K for PEO8k and PCL9k, respectively. The imbibition process was performed under continuous vacuum for a period of 28 days for PEO8k (51 days for PCL9k).

### Differential scanning calorimetry

DSC measurements were made with a Mettler Toledo (DSC-822) calorimeter. Samples (typically ∼5 to 6 mg) were hermetically encapsulated in an aluminum pan. An empty aluminum pan was used as a reference. The melting temperature of the homopolymers was obtained from the second heating run with a rate of 2 K/min. The temperature protocol involved measurements on cooling and subsequent heating in a temperature range between 273 and 363 K. The instrument was calibrated for best performance in the specific temperature range and heating/cooling rate. A precalibration procedure included (i) cleaning of the cell, (ii) a cell-conditioning step (making an inert atmosphere using helium gas), and (iii) an liquid nitrogen cooling system baseline calibration. The main calibration sequence included a baseline calibration using a sapphire standard. In the next step, an indium standard (Δ*H* = 28.71 J/g, *T*_m_ = 428.8 K, with a heating rate of 10 K/min) was used for the enthalpy and melting temperature calibration. As a final step, an empty cell (baseline) measurement verified the successful calibration of the instrument.

### Polarizing optical microscopy

POM measurements were made with an Axioskop 40 FL optical microscope comprising a video camera and a fast frame grabber. Images were taken with the following thermal protocol: First, samples were annealed at *T* = 348 K for 5 min and then quenched at different final temperatures where for isothermal crystallization was followed. The spherulitic growth rates, *G*, were obtained from the linearly growing spherulite diameters with time.

### Scanning electron microscopy

SEM measurements on the fractured surface of AAO templates were performed by LEO Gemini 1530 SEM with an acceleration voltage of 2 kV.

### Atomic force microscopy

The fractured surface of AAO templates with the infiltrated polymers were imaged using a JPK NanoWizard III (JPK Instruments, Berlin) AFM in intermittent contact mode, using a cantilever with a nominal tip radius of 5 to 10 nm, a nominal spring constant of 42 N/m, and a resonance frequency of 352 kHz (OMCL-AC160TS, Olympus, Tokyo). The AFM tip was located above the AAO region of the sample using the top-view video optics of the AFM. In this procedure, the sample was aligned manually to have the pore axis perpendicular to the scan direction of the AFM tip. The obtained phase and height images were processed by Gwyddion software.

### Nano-IR

We performed nano-IR microscopy (VistaScope, Molecular Vista, USA) in the PiFM mode (*44*, *45*). To image the surface topography, we excited the second eigenmode of the cantilever resonance frequency and kept a constant amplitude of vibration with an electronic feedback circuit. The first eigenmode of the cantilever resonance frequency was used to record the IR light induced forces. The incoming IR light was modulated at the difference frequency between the first and second eigenmodes (sideband configuration). This difference frequency was fine-tuned to a maximum response amplitude at the first eigenmode, while the tip was engaged on the surface. Spectra in PiFM mode were recorded by ramping the wave number of the IR light from 850 to 1850 cm^−1^. One spectrum was typically recorded within 30 to 40 s. The data of each spectrum were exported and loaded into Origin (Origin 9.0). We normalized each spectrum by its maximum response amplitude at the first eigenmode. Then, the data were smoothened by a Savitzky-Golay filter (six points). For display reasons, we added an offset to each spectrum.

### Rheology

The viscoelastic behavior of the polymers was measured by a shear rheometer (ARES). Measurements were performed with an environmental test chamber at the respective temperature for imbibition experiments of 330 K (PEO8k) and 323 K (PCL9k). Samples were prepared on the lower plate of the 8- and 25-mm-diameter parallel plate geometry. The storage (*G*′), loss (*G*″) shear moduli and viscosity were monitored as a function of frequency, ω, for frequencies in the range from 10^−2^ < ω < 10^2^ rad/s. The temperature scan on bulk PEO8k sample was made at ω = 10 rad/s following the following thermal protocol: first, by heating the sample above its melting temperature (to *T* = 348 K), followed by slow cooling to 318 K with a rate of 2 K/min. Subsequently, the sample was reheated to the melting temperature.

### Dielectric spectroscopy

Dielectric measurements were performed as a function of frequency for different temperatures using a Novocontrol Alpha frequency analyzer (frequency range from 10^−2^ to 10^7^ Hz). The measurements were obtained on cooling from the melt in 5-K steps. Measurements were carried out in the usual parallel plate geometry with electrodes of 20 mm in diameter. The sample thickness (50 μm) was held constant by poly(tetrafluoroethylene) spacers. The complex dielectric permittivity ε* = ε′ − *i*ε′′, where ε′ is the real and ε′′ is the imaginary part, was obtained as a function of frequency, ω, and temperature, *T*, i.e., ε*(*Τ*, ω). The analysis of the DS curves was based on the empirical equation of Havriliak and Negami (HN)

$$\varepsilon_{\mathrm{HN}}^{*}(T,\omega )-\varepsilon_{\infty }(T)=\frac{\text{Δ}\varepsilon (T)}{{\left\{1+{\left[i\omega \tau_{\mathrm{HN}}(T)\right]}^{m}\right\}}^{n}}$$ (1)

where _∞_(*Τ*) is the high-frequency permittivity, τ_ΗΝ_(*Τ*) is the characteristic relaxation time of the equation, Δε(*Τ*) = ε_ο_(*Τ*) − ε_∞_(*Τ*) is the relaxation strength, *m* and *n* (with limits 0 < *m*, *mn* ≤ 1) describe the symmetrical and asymmetrical broadening of the distribution of relaxation times, respectively. A term describing the conductivity contribution was included in the fitting procedure as σ_ο_(*Τ*)/*i*ε_f_ω, where σ_ο_ is the dc conductivity and ε_f_ is the permittivity of free space. From τ_ΗΝ_, the relaxation times at maximum loss, τ_max_, were obtained analytically from the HN equation as follows

$$\tau_{\max}=\tau_{\mathrm{HN}}\;\sin^{-1/m}\left[\frac{\pi m}{2(1+n)}\right]\sin^{1/m}\left[\frac{\pi mn}{2(1+n)}\right]$$ (2)

These relaxation times correspond to the relaxation times of the segmental process. At lower frequencies, ε′′ rises because of the conductivity [ε′′ = σ/(ωε_f_)]. This conductivity contribution has also been taken into account during the fitting process. Except from the dielectric loss data, the derivative of the real part of the dielectric permittivity, ε′, [dε′/dlnω ≈ (2/π)ε′′] was used in the analysis of the dynamic behavior.

### Small-angle x-ray scattering

SAXS measurements were carried out with a Rigaku MicroMax 007 x-ray generator using Osmic Confocal Max-Flux curved multilayer optics (maximum power of 800 W and brightness of 18 kW/mm^2^ using a Cu target). The detection system was a MAR345 image plate area detector, and the sample-to-detector distance was set at 2.24 m. The samples (thickness, ~1 mm) used in SAXS were prepared with different cooling rates (1, 5, 10, and 20 K/min for PEO8k; 1 and 20 K/min for PCL9k) with a Linkam temperature control unit (THMS600) with built-in TMS94 temperature programmer. The recorded intensity distributions were integrated along the equatorial and meridional axes and are presented as a function of the modulus of the scattering vector, *q* = (4π/λ) sin(2θ/2), where λ (=1.54184 nm) is the wavelength and 2θ is the scattering angle. Measurements of 3600 s long were made at different temperatures.

## Supplementary Materials

### This PDF file includes:

Supplementary Text Figs. S1 to S11 Tables S1 and S2 References
