# Supplementary material for: When crystals flow
Source: Sci Adv. 2023 May 10;9(19):eadg8865. doi: 10.1126/sciadv.adg8865 (PMC10171800; doi:10.1126/sciadv.adg8865)
Supplement: Supplementary file 1 — Supplementary Text Figs. S1 to S11 Tables S1 and S2 References [file sciadv.adg8865_sm.pdf]

Science Advances
AAAS

Supplementary Materials for

When crystals flow

Chien-Hua Tu *et al.*

Corresponding author: George Floudas, gfloudas@uoi.gr

Sci. Adv. **9**, eadg8865 (2023)
DOI: 10.1126/sciadv.adg8865

This PDF file includes:

Supplementary Text
Figs. S1 to S11
Tables S1 and S2
References

Supplementary Text

Fig. S1 provides the nano IR response of a semicrystalline PEO8k film. The film was slowly crystallized from the melt. Absorption bands at 1149, 1100 and 1061 cm^{-1} are characteristic of the semicrystalline PEO. In the case of amorphous PEO these absorption bands become very broad and appear as a single asymmetric broad band (*46*).

Fig. S2 is a composite plot that provides the thermal (a), the mechanical (b), the SAXS (c) and the optical (d) characterization of semicrystalline PCL9k. The DSC heating curve (Fig. S2A) shows a melting peak at a characteristic temperature of 328 K (degree of crystallinity ~ 49%). The temperature of imbibition was at 323 K. At the imbibition temperature the mechanical response is purely elastic with frequency independent moduli and G'>G" (Fig. S2B). The domain spacing of PCL9k, obtained from SAXS (Fig. S2C), is about 12.8 nm, which is far smaller than the pore size (400 nm). The POM image (Fig. S2D) shows PCL spherulites with a very high density.

Fig. S3 shows the AFM image of the PEO8k film on top of AAO template revealing the long fibril structure.

After 51-days of imbibition at 323 K we employed SEM and AFM to characterize the cross-section of the AAO templates. Fig. S4A, left depicts a usual SEM picture and Fig. S4A, right one taken via high-angle back scattering electrons, that can provide a better contrast between the infiltrated and empty regions. Fig. S4B shows the AFM phase image from a cross-section of the AAO template taken in a pre-selected area according to the SEM image of Fig. S4A.

Fig. S5 show the structure of the PCL9k film on top the AAO template and the corresponding periodicity of ~ 15 nm, *i.e.*, in agreement with the one periodicity of the lamellar structure estimated from SAXS.

Fig. S6 depicts the viscoelastic properties of PEO8k recorded at a single frequency of ω=10 rad/s by cooling and subsequent heating.

Fig. S7 provides the dielectric relaxation frequencies corresponding to the segmental process of PEO and PCL as a function of inverse temperature. The effect of confinement is to speed-up the segmental process and to lower the liquid-to-glass temperature of PEO and PCL. However, such relatively small changes in T_g cannot account for the enhanced imbibition lengths.

Fig. S8 shows the dielectric relaxation frequencies corresponding to the segmental process of PEO 5k plotted as a function of inverse temperature. The line is a fit to the VFT equation. The relaxation times in the high temperature region are obtained by extrapolation.

Fig. S9 depicts the measured spherulitic growth rates of PEO 8k and the calculated τ_{lc} as a function of temperature.

Fig. S10 shows the DSC heating curve and the SEM micrograph of PEO 1k and 500k reveals the fast imbibition of PEO 1k and extremely slow imbibition of PEO 500k crystals into AAO 400 nm nanopores.

Fig. S11 addresses the question of the importance of capillarity in the flow of polymer crystals. To address this question a PEO 8k film was placed on top an electropolished alumina surface at temperatures below the melting point for a period of 28 days (*i.e.*, conditions similar to the imbibition experiments in nanopores). The AFM measurement results reveal the absence of any

adsorption by the polymer chains on the alumina next to the polymer film. This result demonstrates the importance of capillarity in the observed flow of semicrystalline polymers in nanopores.

Table S1 shows the molecular characteristics for the polymers used in this study.

Table S2 shows the original data plotted in Fig. 4c in the main text.

Table S1. Molecular characteristics of the PEO and PCL homopolymers employed in this study.

Sample	M_n (g·mol^{-1})	M_w (g·mol^{-1})	*Đ*	T_m (K)	ΔH_m [a]	X_c (%)[b]
PEO1k	825	1011	1,23	311	13	6
PEO8k	8090	9155	1,13	335	139	70
PEO500k	398000	480000	1,21	341	121	61
PCL9k	5366	9723	1,81	329	72	50

[a] Heat of fusion determined from the second heating run in the DSC thermograph.
[b]The degree of crystallinity (X_c) was evaluated based on the heat of fusion as X_c (%)=($\Delta H_m / \Delta H^o_m$)×100%. Here, ΔH^o_m represents the heat of fusion of the "ideal" crystal (*47*) (197.8 J/g for linear PEO and 142.9 J/g for linear PCL).

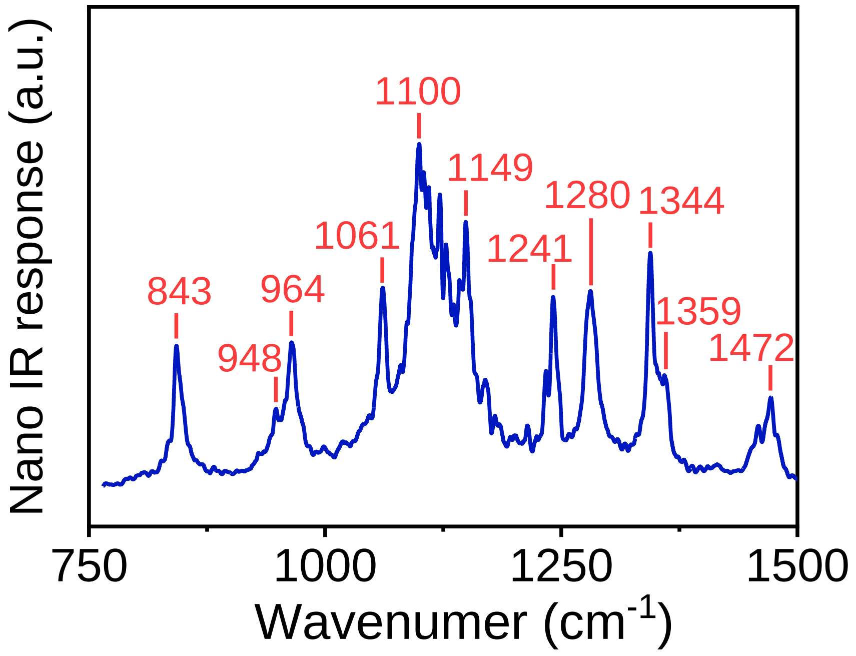

Fig. S1. Nano IR of semicrystalline PEO. Nano IR spectrum of a semicrystalline PEO film (PEO 8k) deposited on a glass slide. All major peaks that are present in an absorbance measurement are visible in the nano IR measurement as well. In particular, the region between 990 cm^{-1} and 1200 cm^{-1} is characteristic for CH_2 and CO rocking/stretching vibrations of semi-crystalline PEO with three noticeable absorption peaks at 1149, 1100 and 1061 cm^{-1}.

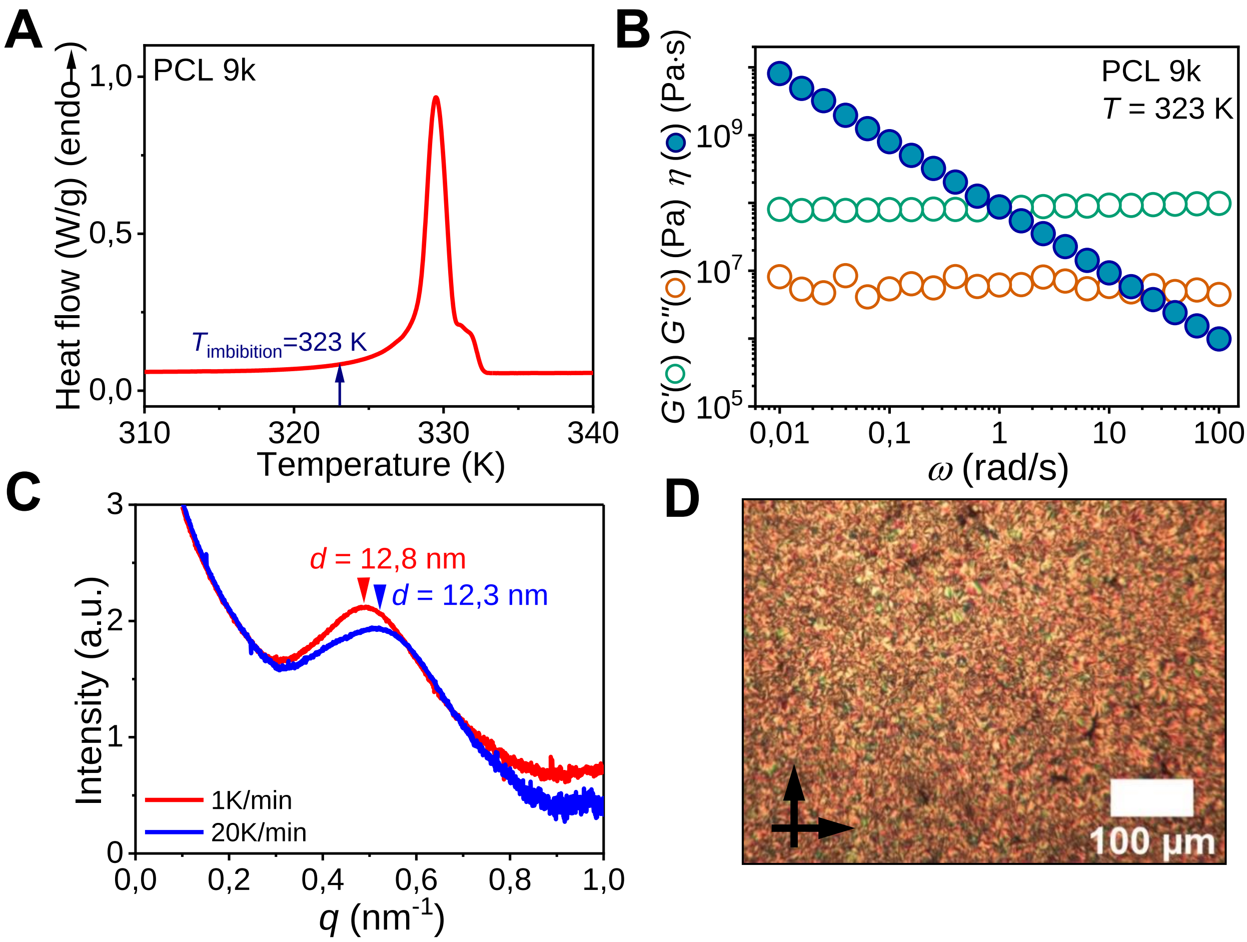

Fig. S2. Thermodynamic, rheological and crystallization behavior of bulk PCL9k. (A) DSC heating curve (rate 2 K/min) with the indicated imbibition temperature at 323 K. (B) Frequency dependence of the storage modulus, the loss modulus and of the viscosity measured at the imbibition temperature of 323 K. (C) SAXS curves taken at ambient temperature following cooling from the melt by two different cooling rates. The respective domain spacings are shown. (D) Spherulitic morphology depicting a high nucleation density obtained at ambient temperature following cooling from the melt.

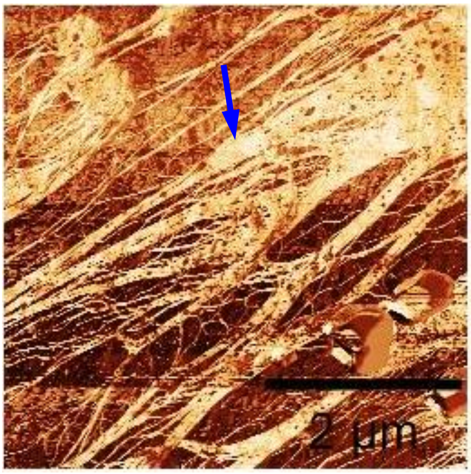

Fig. S3. AFM of semicrystalline PEO. AFM phase image of PEO8k film deposited on top the AAO template used in the infiltration experiments. The blue arrow indicates the long fibrilar structure. The bar indicates a length of 2 μm.

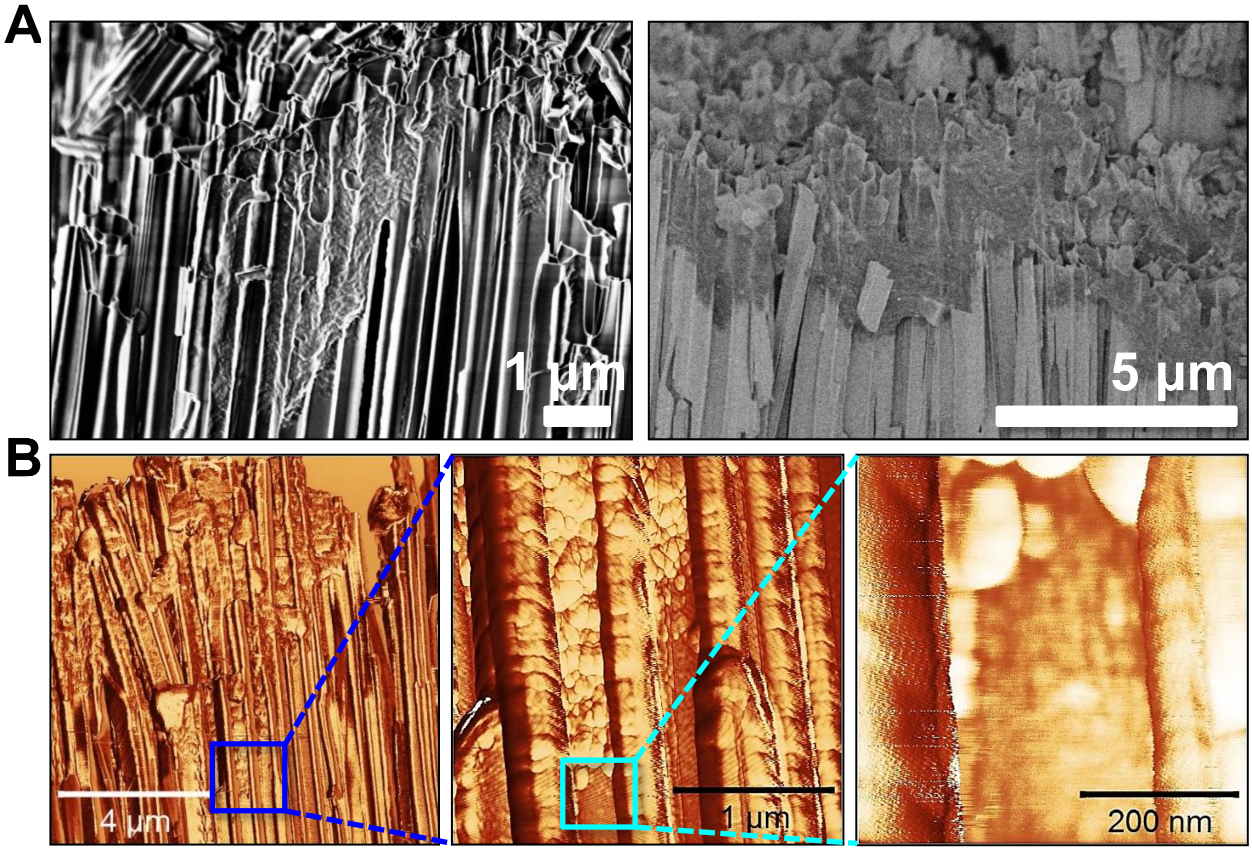

Fig. S4. Imbibition of PCL 9k in nanopores revealed by SEM and AFM. (A) (left) Usual SEM and (right) high-angle back scattering electron (HA-BSE) SEM images obtained from cross-sections of an AAO template having pores of 400 nm in diameter. The template was infiltrated with PCL9k at 323 K for 51days. (B) (left) AFM 2D phase image to the same region as (A), (middle) zoom-in to the blue squared dashed area in (B, left), and (right) zoom-in to the cyan squared dashed area in (B, middle).

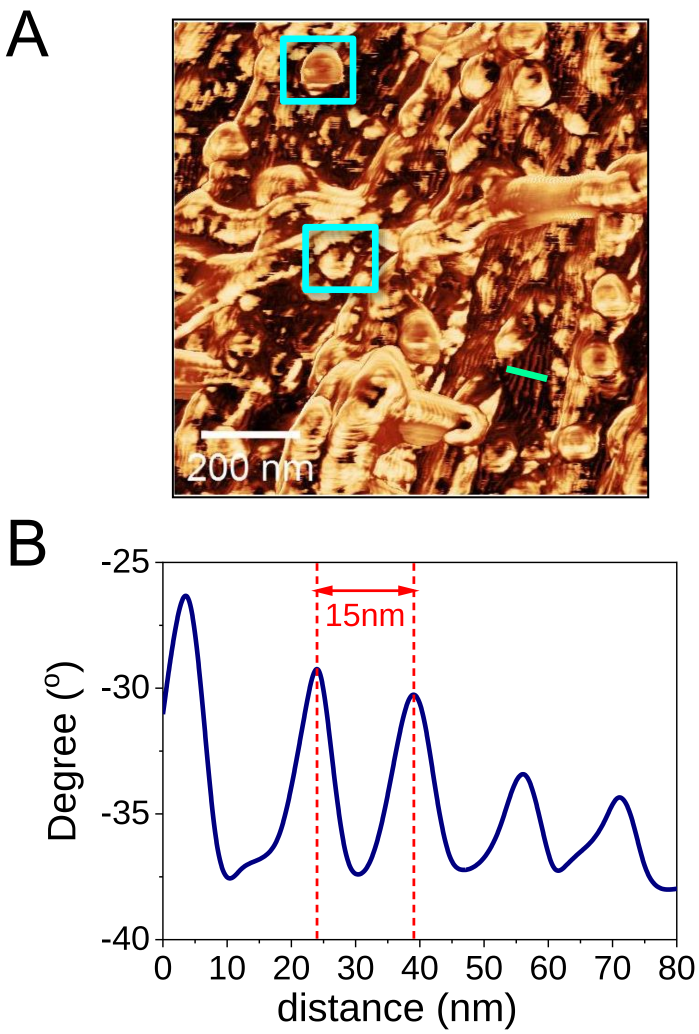

Fig. S5. Lamellar structure of PCL on top AAO revealed by AFM. (A) AFM phase image of PCL9k film deposited on top the AAO template. The cyan squared area indicates the grain structure. (B) The profile along the green line from the left image, reveals a lamellar structure with a periodicity of about 15 nm.

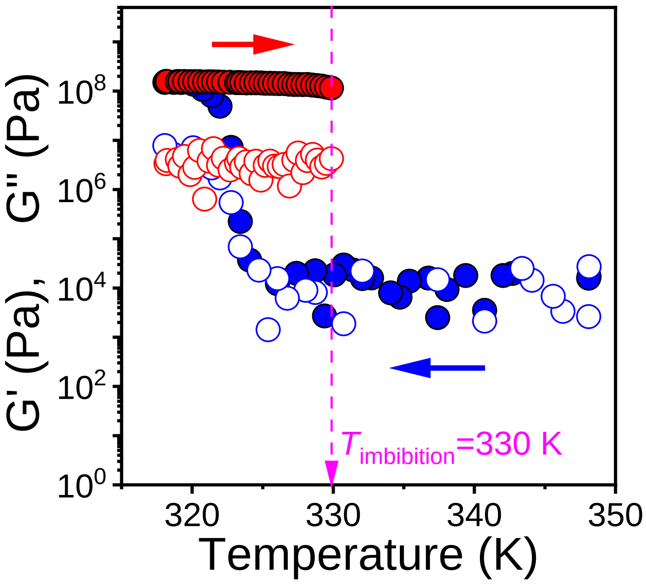

Fig. S6. Viscoelastic properties of PEO in the bulk. Viscoelastic properties of PEO8k in bulk as a function of temperature under isochronal (ω=10 rad/s) conditions. Storage modulus, G', (filled symbols) and loss modulus G'' (open symbols) are shown during cooling (blue) and subsequent heating (red). The imbibition temperature is indicated with the vertical arrow.

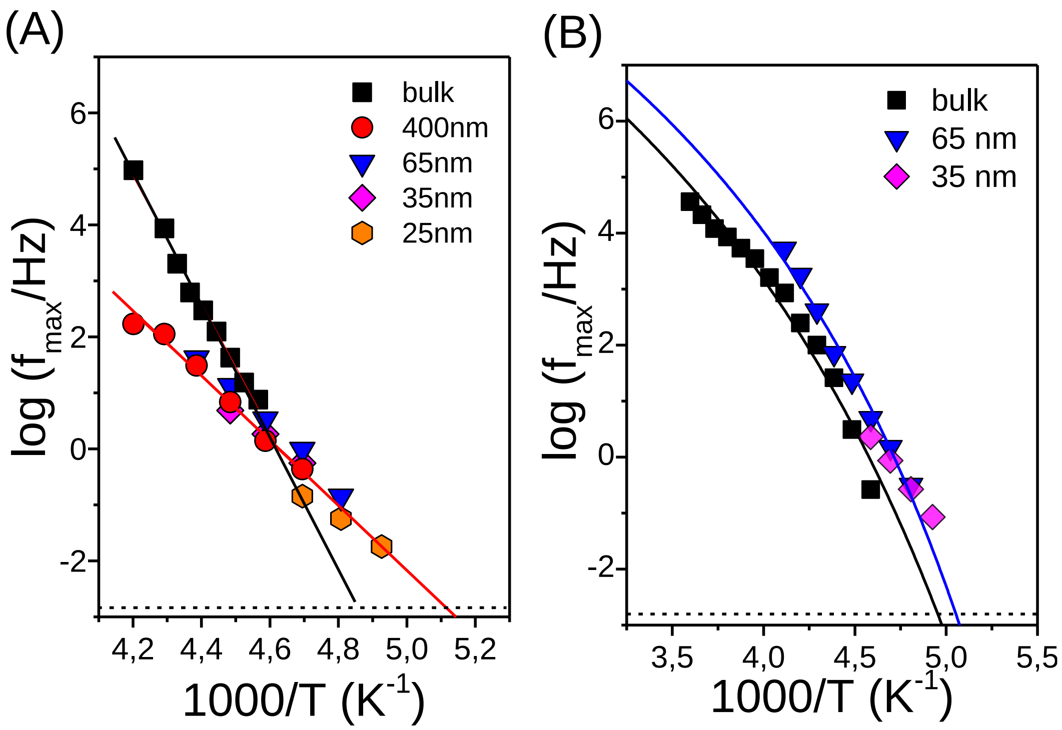

Figure S7. Segmental dynamics of confined PEO and PCL. Characteristic relaxation frequencies corresponding to the dielectric loss peak of the segmental relaxation of (**A**) PEO and (**B**) PCL confined within AAO nanopores and plotted versus inverse temperature. The horizontal dashed line gives the characteristic peak frequency at T_g (f_{max}=1/2$\pi\tau_{max}$, τ_{max}=100 s). Lines in (A) depict fits to an Arrhenius equation. Lines in (B) represent fits to the Vogel-Fulcher-Tammann equation.

Estimation of the different characteristic times in Figure 4C (main text)

In the following we describe the characteristic times listed in Fig. 4c in the main text.

- $\boldsymbol{\tau_{\text{segmental}}}$ – The temperature dependence of the segmental relaxation times, $\tau_{\text{segmental}}$, of PEO was extracted by extrapolating the Vogel-Fulcher-Tammann (VFT) equation of bulk PEO 5k data (blue solid triangles in **Fig. S7**; measured by dielectric spectroscopy, to the higher temperature region (blue empty triangles in **Fig. S7**).

- **<$\boldsymbol{\tau_c}$>** – The <τ_c>(T) is obtained by the following equation (*26,48,49*):

$$\langle\tau_c\rangle = \tau_0 \cdot \left(\frac{d_c}{4{,}6\;nm} + 0{,}77\right) exp(\frac{E_a}{RT})$$

Here, τ_0 = 8·10^{-18} s and the activation energy is E_a = 64.5 kJ·mol^{-1}. The value of d_c is fixed at 11,02 nm.

- $\boldsymbol{\tau_{\text{stem}}}$ – The temperature dependence of τ_{stem} is derived from <τ_c>(T) as follows (*26,48,49*):

$$\tau_{stem} \approx \langle\tau_c\rangle \cdot \frac{d_c^2}{\Delta z_c^2}$$

Here, Δz_c is the length along c-axis of crystal unit cell with a value of 0.279 nm for 7_2 helix structure of PEO.

- $\boldsymbol{\tau_{\text{lc}}}$ – The temperature dependence of τ_{lc} is obtained as follows (*26,48,49*):

$$\tau_{lc} = \frac{l\;(nm)}{G\;(\frac{\mu m}{s})} = \frac{\left\langle\frac{a+b}{2}\right\rangle}{G\;(T)}$$

Here, l is the intermolecular distance (or the distance between unit crystals) given by the average value over the a and b axis of PEO crystal unit cell is $\left\langle\frac{a+b}{2}\right\rangle = \left\langle\frac{0{,}8+1{,}3}{2}\right\rangle$=1.05 nm. G represents the growth rate of spherulites obtained by recording the isothermal crystallization of PEO at different temperatures (i.e. from 320 K to 331 K, in 1 K steps) under polarized optical microscopy. The thus obtained $\tau_{\text{lc}}(T)$ and $G(T)$ are displayed in Figure S9.

- $\boldsymbol{\tau_{\text{adsorption}}}$ – The $\tau_{\text{adsorption}}$ is adopted from our confinement study of a PEO-based polymer electrolyte with same molar mass of PEO but within a different pore size (100 nm in that study) at 303 K (*36*).

Table S2. Characteristic times employed in Fig. 4 (main text).

Temperature (K)	$\tau_{segmental}$/s	$<\tau_c>$/s	τ_{stem}/s	τ_{lc}/s	$\tau_{adsorption}$/s (at 303 K)
318	$2,6\times10^{-7}$	$9,8\times10^{-7}$	$1,5\times10^{-3}$	-	$2,34\times10^{4}$
319	$2,4\times10^{-7}$	$9,1\times10^{-7}$	$1,4\times10^{-3}$	-	-
320	$2,2\times10^{-7}$	$8,4\times10^{-7}$	$1,3\times10^{-3}$	$1,1\times10^{-4}$	-
321	$2,1\times10^{-7}$	$7,8\times10^{-7}$	$1,2\times10^{-3}$	$1,8\times10^{-4}$	-
322	$1,9\times10^{-7}$	$7,2\times10^{-7}$	$1,1\times10^{-3}$	$3,1\times10^{-4}$	-
323	$1,7\times10^{-7}$	$6,7\times10^{-7}$	$1,0\times10^{-3}$	$5,4\times10^{-4}$	-
324	$1,6\times10^{-7}$	$6,2\times10^{-7}$	$9,7\times10^{-4}$	$1,5\times10^{-3}$	-
325	$1,5\times10^{-7}$	$5,8\times10^{-7}$	$9,0\times10^{-4}$	$3,6\times10^{-3}$	-
326	$1,4\times10^{-7}$	$5,4\times10^{-7}$	$8,4\times10^{-4}$	$1,0\times10^{-2}$	-
327	$1,3\times10^{-7}$	$5,0\times10^{-7}$	$7,8\times10^{-4}$	$2,0\times10^{-2}$	-
328	$1,2\times10^{-7}$	$4,6\times10^{-7}$	$7,3\times10^{-4}$	$1,0\times10^{-1}$	-
329	$1,1\times10^{-7}$	$4,3\times10^{-7}$	$6,8\times10^{-4}$	$4,3\times10^{-1}$	-
330	$1,0\times10^{-7}$	$4,0\times10^{-7}$	$6,3\times10^{-4}$	1,4	-
331	$9,8\times10^{-8}$	$3,7\times10^{-7}$	$5,9\times10^{-4}$	3,0	-
332	$9,2\times10^{-8}$	$3,5\times10^{-7}$	$5,5\times10^{-4}$	-	-
333	$8,6\times10^{-8}$	$3,2\times10^{-7}$	$5,1\times10^{-4}$	-	-

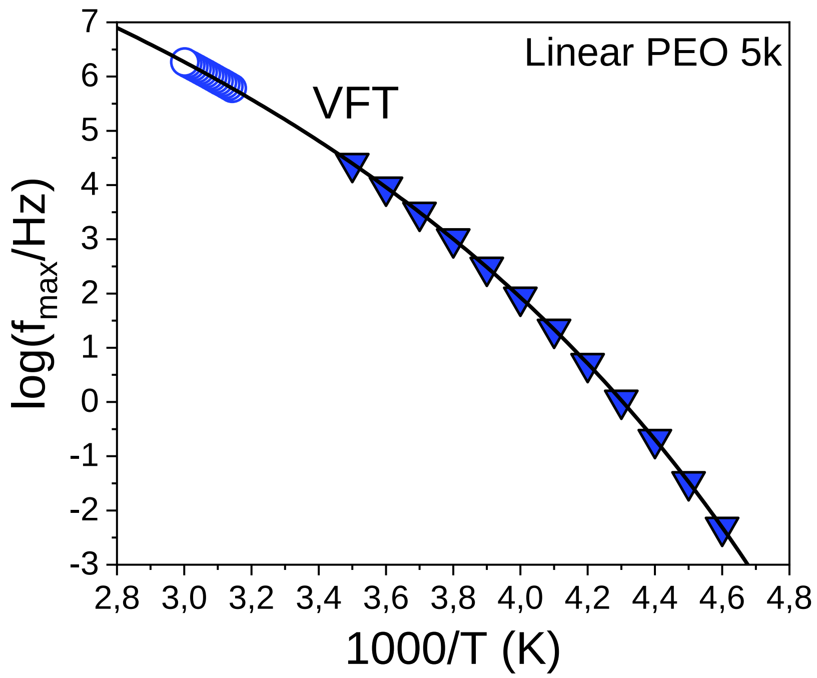

Figure S8. Segmental dynamics of PEO 5k as a function of inverse temperature. Temperature dependence of the characteristic frequencies associated with the segmental process (α-relaxation) for a linear PEO 5k: (blue solid triangles) (*33*). The empty blue circles indicate the segmental dynamics by extrapolation to higher temperatures. The black solid line is a fit to the VFT equation with parameters: $\tau_0 = 10^{-12}$ s, B = 2550 K, and T_0 = 140 K.

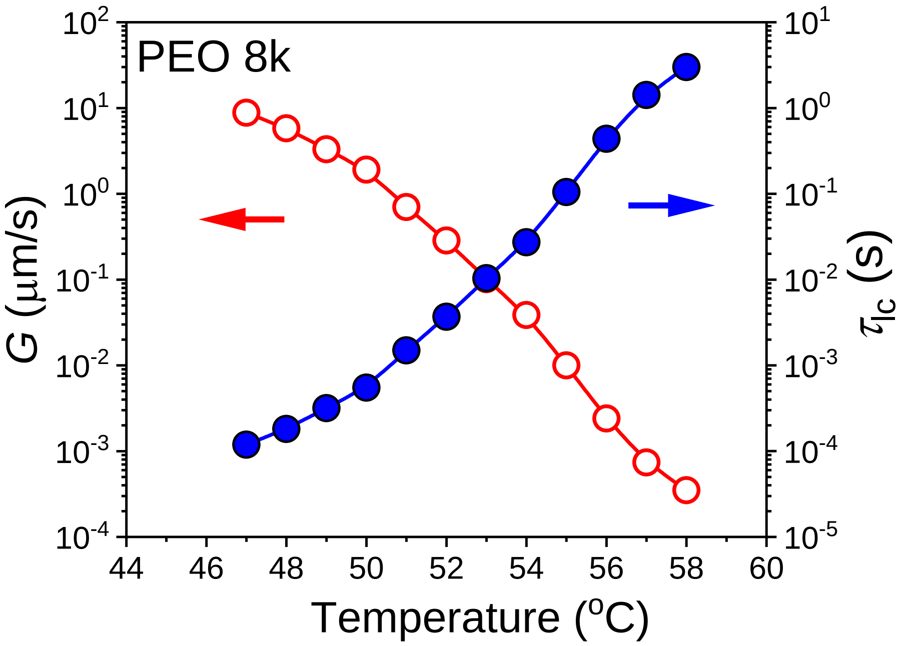

Figure S9. Spherulitic growth rates for PEO 8k. Temperature dependence of spherulitic growth rates of PEO8k obtained under isothermal crystallization (red circles) and the calculated τ_{lc} (blue solid circles).

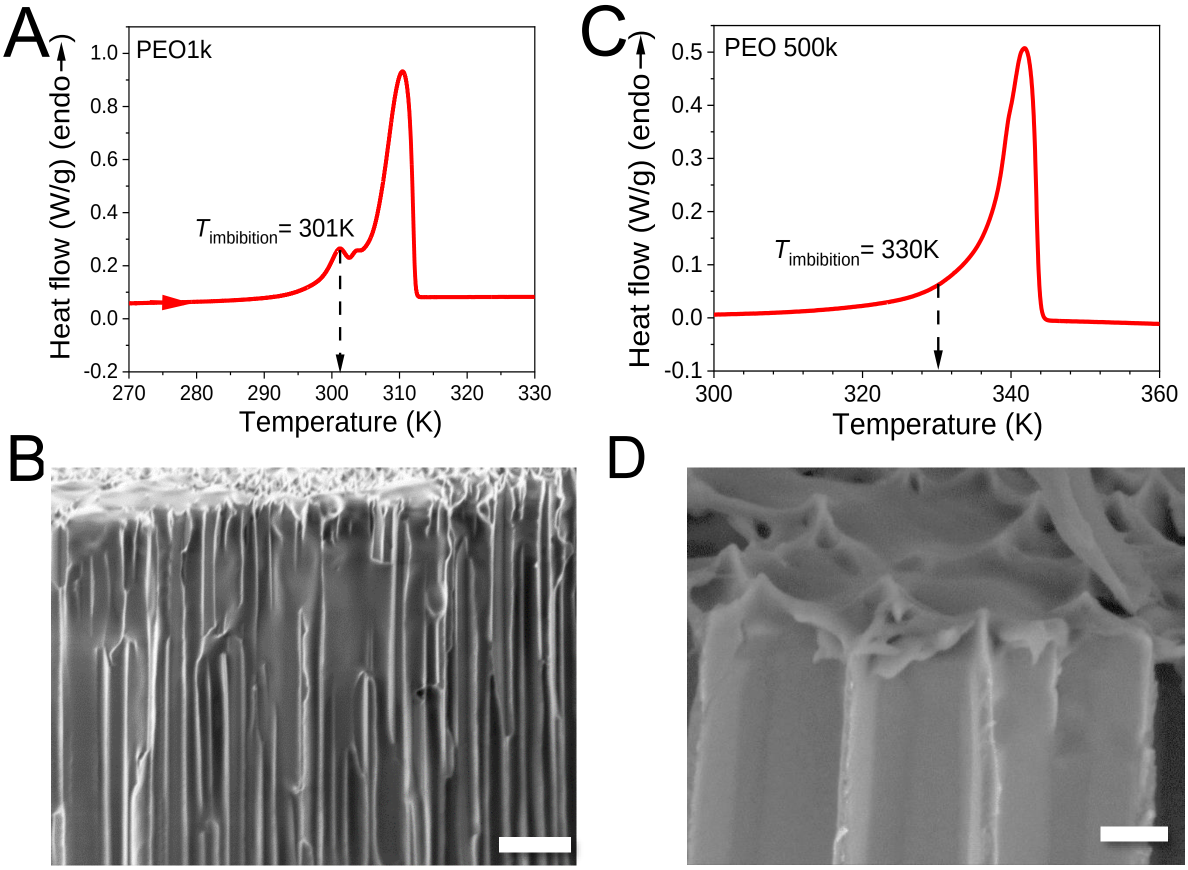

Figure S10. Dependence of the imbibition length on polymer molar mass. (A) DSC heating curve of bulk PEO1k showing the main melting peak at 310 K. Imbibition is this case was made at T = 301 K. (B) SEM micrograph from a cross-section of PEO 1k located inside AAO with pore diameter of 400 nm obtained at T = 301 K after a period of 21 days. The pores are fully infiltrated. (C) DSC heating curve of bulk PEO500k showing the main melting peak at 342 K. Imbibition in this case was made at T = 330 K. (D) SEM micrograph from a cross-section of a AAO template with 400 nm pores infiltrated by PEO 500k at T = 330 K for a period of 35 days. The micrograph depicts empty pores; chains could not enter the pores for this high molar mass polymer. The white scale bar in (B) and (D) indicates 2 μm and 200 nm, respectively.

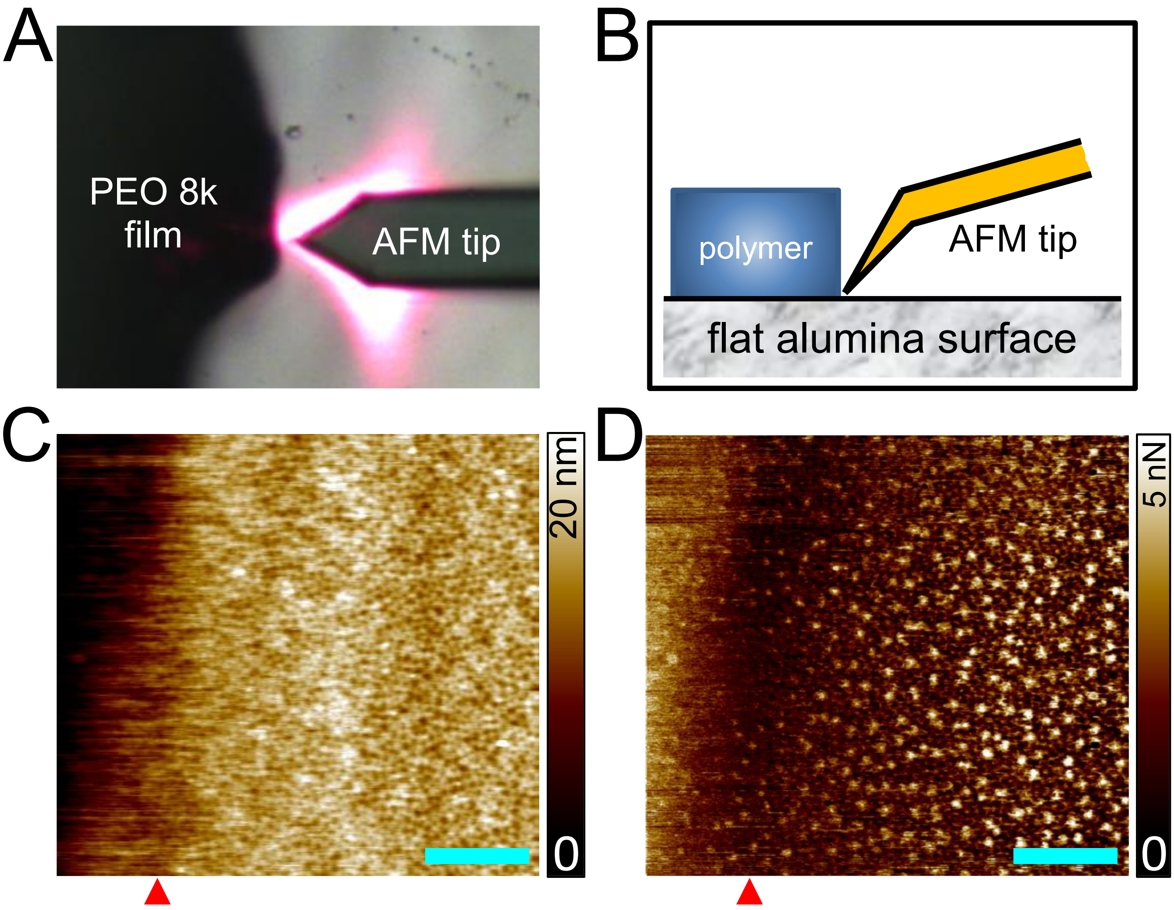

Figure S11. Is capillarity important for polymer imbibition in nanopores? A PEO8k film deposited on top of a flat electropolished alumina surface at $T = 330$ K (*i.e.*, well below the melting temperature) for period of 28 days is measured by AFM. Real-time experiment image (A) and schematic (B) showing that the AFM tip explores an area in close proximity to the edge of the PEO film. The measured 2D AFM topography image (C) and the corresponding adhesion image (D) depicts a polymer-free alumina surface in an area close to the film edge (indicated by the red arrow). There is absence of adsorption of a flat alumina surface by the polymer film. This is a clear manifestation that capillarity plays a major role in the adsorption mechanism. The cyan scale bar indicates a length of 1 µm.

REFERENCES AND NOTES

1. W. Rosenhain, D. Ewen, The intercrystalline cohesion of metals (second paper). *J. Inst. Met.* **10**, 119–149 (1913).

2. M. Chandross, N. Argibay, Friction of metals: A review of microstructural evolution and nanoscale phenomena in shearing contacts. *Tribology Lett.* **69**, 119 (2021).

3. H. Zhang, D. J. Srolovitz, J. F. Douglas, J. A. Warren, Grain boundaries exhibit the dynamics of glass-forming liquids. *Proc. Natl. Acad. Sci. U.S.A.* **106**, 7735–7740 (2009).

4. A. B. Belonoshko, T. Lukinov, J. Fu, J. Zhao, S. Paris, S. I. Simak, Stabilization of body-centred cubic iron under inner-core conditions. *Nat. Geosci.* **10**, 312–316 (2017).

5. M. Millot, S. Hamel, J. R. Rygg, P. M. Celliers, G. W. Collins, F. Coppari, D. E. Fratanduono, R. Jeanloz, D. C. Swift, J. H. Eggert, Experimental evidence for superionic water ice using shock compression. *Nat. Phys.* **14**, 297–302 (2018).

6. M. Faraday, Lecture given at Royal Institution, Athenaeum (London, England, 1850), pp. 640–641.

7. R. Merkle, J. Maier, Z. Anorg, On the Tammann–Rule. *Allg. Chem.* **631**, 1163–1166 (2005).

8. X. Wang, X. Tong, H. Zhang, J. F. Douglas, String-like collective motion and diffusion in the interfacial region of ice. *J. Chem. Phys.* **147**, 194508 (2017).

9. G. Materzanini, T. Chiarotti, N. Marzari, Solids that are also liquids: Elastic tensors of superionic materials. *NPJ Comput. Mater*. **9**, 10 (2023).

10. H. Zhang, H. Wang, J. F. Douglas, Localization model description of diffusion and structural relaxation in superionic crystalline UO_2. *J. Chem. Phys.* **151**, 071101 (2019).

11. J. Fu, Superionic conductivity of glass-ceramics in the system Li_2O-Al_2O_3-TiO_2-P_2O_5. *Solid State Ion.* **96**, 195–200 (1997).

12. S. D. Jones, H. Nguyen, P. M. Richardson, Y.-Q. Chen, K. E. Wyckoff, C. J. Hawker, R. J. Clément s, G. H. Fredrickson, R. A. Segalman, Design of polymeric Zwitterionic solid electrolytes with superionic lithium transport. *ACS Cent. Sci.* **8**, 169–175 (2022).

13. G. Strobl, *The Physics of Polymers: Concepts for Understanding their Structures and Behavior* (Springer, 2007).

14. X. Tang, W. Chen, L. Li, The tough journey of polymer crystallization: Battling with chain flexibility and connectivity. *Macromolecules* **52**, 3575–3591 (2019).

15. H.-N. Lee, K. Paeng, S. F. Swallen, M. D. Ediger, Direct measurement of molecular mobility in actively deformed polymer glasses. *Science* **323**, 231–234 (2009).

16. K. Shin, S. Obukhov, J.-T. Chen, J. Huh, Y. Hwang, S. Mok, P. Dobriyal, P. Thiyagarajan, T. P. Russell, Enhanced mobility of confined polymers. *Nat. Mater.* **6**, 961–965 (2007).

17. A. Johner, K. Shin, S. Obukhov, Nanofluidity of a polymer melt: Breakdown of Poiseuille's flow model. *Europhys. Lett.* **91**, 38002 (2010).

18. J. F. Douglas, H. E. Johnson, S. Granick, A simple kinetic model of polymer adsorption and desorption. *Science* **262**, 2010–2012 (1993).

19. S. Granick, Motion and relaxation of confined liquids. *Science* **253**, 1374–1379 (1991).

20. Y. Yao, S. Alexandris, F. Henrich, G. Auernhammer, M. Steinhart, H.-J. Butt, G. Floudas, Complex dynamics of capillary imbibition of poly(ethylene oxide) melts in nanoporous alumina. *J. Chem. Phys.* **146**, 203320 (2017).

21. H. Masuda, K. Fukuda, Ordered metal nanohole arrays made by a two-step replication of honeycomb structures of anodic alumina. *Science* **268**, 1466–1468 (1995).

22. M. Steinhart, Supramolecular organization of polymeric materials in nanoporous hard templates. *Adv. Polym. Sci.*, **220**, 123–187 (2008).

23. H. Duran, M. Steinhart, H.-J. Butt, G. Floudas, From heterogeneous to homogeneous nucleation of isotactic poly(propylene) confined to nanoporous alumina. *Nano Lett.* **11**, 1671–1675 (2011).

24. Y. Suzuki, M. Steinhart, M. Kappl, H.-J. Butt, G. Floudas, Effects of polydispersity, additives, impurities and surfaces on the crystallization of poly (ethylene oxide)(PEO) confined to nanoporous alumina. *Polymer* **99**, 273–280 (2016).

25. P. Huber, Soft matter in hard confinement: Phase transition thermodynamics, structure, texture, diffusion and flow in nanoporous media. *J. Phys. Condens. Matter* **27**, 103102 (2015).

26. M. Schulz, M. Schäfer, K. Saalwächter, T. Thurn-Albrecht, Competition between crystal growth and intracrystalline chain diffusion determines the lamellar thickness in semicrystalline polymers. *Nat. Commun.* **13**, 119 (2022).

27. C. Bergeron, E. Perrier, A. Potier, G. Delmas, *International Journal of Spectroscopy* (Hindawi Publishing Corporation, 2012), Article ID 432046.

28. R. Lucas, Ueber das Zeitgesetz des kapillaren Aufstiegs von Flüssigkeiten. *Kolloid-Zeitschrift* **23**, 15–22 (1918).

29. E. W. Washburn, The dynamics of capillary flow. *Phys. Rev.* **17**, 273–283 (1921).

30. S. Alexandris, P. Papadopoulos, G. Sakellariou, M. Steinhart, H.-J. Butt, G. Floudas, Interfacial energy and glass temperature of polymers confined to nanoporous alumina. *Macromolecules* **49**, 7400–7414 (2016).

31. Y. Yao, H.-J. Butt, G. Floudas, J. Zhou M. Doi, Theory on capillary filling of polymer melts in nanopores. *Macromol. Rapid Commun.* **39**, 1800087 (2018).

32. K. Liu, Y. Song, W. Feng, N. Liu, W. Zhang, X. Zhang, Extracting a single polyethylene oxide chain from a single crystal by a combination of atomic force microscopy imaging and single-molecule force spectroscopy: Toward the investigation of molecular interactions in their condensed states. *J. Am. Chem. Soc.* **133**, 3226–3229 (2011).

33. Y. Suzuki, H. Duran, M. Steinhart, H.-J. Butt, G. Floudas, Homogeneous crystallization and local dynamics of poly(ethylene oxide) (PEO) confined to nanoporous alumina. *Soft Matter* **9**, 2621–2628 (2013).

34. M. Schulz, A. Seidlitz, R. Kurz, R. Barenwald, A. Petzold, K. Saalwächter, T. Thurn-Albrecht, The underestimated effect of intracrystalline chain dynamics on the morphology and stability of semicrystalline polymers. *Macromolecules* **51**, 8377–8385 (2018).

35. C.-H. Tu, J. Zhou, H.-J. Butt, G. Floudas, Adsorption kinetics of cis-1,4-Polyisoprene in nanopores by in situ nanodielectric spectroscopy. *Macromolecules* **54**, 6267–6274 (2021).

36. C.-H. Tu, L. Veith, H.-J. Butt, G. Floudas, Ionic conductivity of a solid polymer electrolyte confined in nanopores. *Macromolecules* **55**, 1332–1341 (2022).

37. W. I. Edens, C. B. Hebert, U.S. Patent US007105021B2 (2006).

38. A. Serghei, W. Zhao, D. Miranda, T. P. Russell, Curie transitions for attograms of ferroelectric polymers. *Nano Lett.* **13**, 577–580 (2013).

39. A. Pipertzis, K. Asadi, G. Floudas, P(VDF-TrFE) copolymer dynamics as a function of temperature and pressure in the vicinity of the curie transition. *Macromolecules* **55**, 2746–2757 (2022).

40. M. M. Abolhasani, M. Naebe, K. Shirvanimoghaddam, H. Fashandi, H. Khayyam, M. Joordens, A. Pipertzis, S. Anwar, R. Berger, G. Floudas, J. Michels, K. Asadi, Thermodynamic approach to tailor porosity in piezoelectric polymer fibers for application in nanogenerators. *Nano Energy* **62**, 594–600 (2019).

41. H. Masuda, K. Fukuda, Ordered metal nanohole arrays made by a two-step replication of honeycomb structures of anodic alumina. *Science* **268**, 1466–1468 (1995).

42. H. Masuda, F. Hasegawa, S. Ono, Self-ordering of cell arrangement of anodic porous alumina formed in sulfuric acid solution. *J. Electrochem. Soc.* **144**, L127–L130 (1997).

43. H. Masuda, K. Yada, A. Osaka, Self-ordering of cell configuration of anodic porous alumina with large-size pores in phosphoric acid solution. *Jpn. J. Appl. Phys.* **37**, L1340 (1998).

44. D. Nowak, W. Morrison, H. K. Wickramasinghe, J. Jahng, E. Potma, L. Wan, R. Ruiz, T. R. Albrecht, K. Schmidt, J. Frommer, D. P. Sanders, S. Park, Nanoscale chemical imaging by photoinduced force microscopy. *Sci. Adv.* **2**, e1501571 (2016).

45. R. A. Murdick, W. Morrison, D. Nowak, T. R. Albrecht, J. Jahng, S. Park, Photoinduced force microscopy: A technique for hyperspectral nanochemical mapping. *Jpn. J. Appl. Phys.* **56**, 08LA04 (2017).

46. C. Bergeron, E. Perrier, A. Potier, G. Delmas, A study of the deformation, network, and aging of polyethylene oxide films by infrared spectroscopy and calorimetric measurements. *Int. J. Spectrosc.* **2012**, 432046 (2012).

47. J. E. Mark, Ed., *Physical Properties of Polymers Handbook* (Springer, ed. 2, 2007).

48. R. Kurz, A. Achilles, W. Chen, M. Schäfer, A. Seidlitz, Y. Golitsyn, J. Kressler, W. Paul, G. Hempel, T. Miyoshi, T. Thurn-Albrecht, K. Saalwächter, Intracrystalline jump motion in poly(ethylene oxide) lamellae of variable thickness: A comparison of NMR methods. *Macromolecules* **50**, 3890–3902 (2017).

49. M. Schulz, A. Seidlitz, R. Kurz, R. Bärenwald, A. Petzold, K. Saalwächter, T. Thurn-Albrecht, The underestimated effect of intracrystalline chain dynamics on the morphology and stability of semicrystalline polymers. *Macromolecules* **51**, 8377–8385 (2018).

50. M. Schulz, M. Schäfer, K. Saalwächter, T. Thurn-Albrecht, Competition between crystal growth and intracrystalline chain diffusion determines the lamellar thickness in semicrystalline polymers. *Nat. Commun.* **13**, 119 (2022).

51. G. Zardalidis, J. Mars, J. Allgaier, M. Mezger, D. Richter, G. Floudas, Influence of chain topology on polymer crystallization: Poly(ethylene oxide) (PEO) rings vs. linear chains. *Soft Matter* **12**, 8124–8134 (2016).
